# A Dynamic Mobile DNA Family in the Yeast Mitochondrial Genome

**DOI:** 10.1534/g3.115.017822

**Published:** 2015-04-20

**Authors:** Baojun Wu, Weilong Hao

**Affiliations:** Department of Biological Sciences, Wayne State University, Detroit, Michigan 48202

**Keywords:** GC cluster, horizontal transfer, mitochondrial genome, transposable element, palindromic sequence

## Abstract

Transposable elements (TEs) are an important factor shaping eukaryotic genomes. Although a significant body of research has been conducted on the abundance of TEs in nuclear genomes, TEs in mitochondrial genomes remain elusive. In this study, we successfully assembled 28 complete yeast mitochondrial genomes and took advantage of the power of population genomics to determine mobile DNAs and their propensity. We have observed compelling evidence of GC clusters propagating within the mitochondrial genome and being horizontally transferred between species. These mitochondrial TEs experience rapid diversification by nucleotide substitution and, more importantly, undergo dynamic merger and shuffling to form new TEs. Given the hyper mobile and transformable nature of mitochondrial TEs, our findings open the door to a deeper understanding of eukaryotic mitochondrial genome evolution and the origin of nonautonomous TEs.

Transposable elements (TEs) are widely distributed among eukaryotic nuclear genomes and are a major contributor to genomic variation (Collier and Largaespada 2007; Deragon *et al.* 2008). In contrast to our detailed understanding of nuclear TEs, TEs in the organelle genomes are less well-characterized. Several TE-like sequences have been reported in plant and yeast mitochondrial (mt)DNA; however, all these TE-like sequences are fragments of TEs from the nuclear genome that have arisen from nuclear-derived insertions (Knoop *et al.* 1996; Alverson *et al.* 2010; Rodriguez-Moreno *et al.* 2011; Mularoni *et al.* 2012; Islam *et al.* 2013).

Another kind of mitochondrial TE-like elements are palindromic GC clusters, which are characterized by their relatively high GC content and palindromic structure (Yin *et al.* 1981; de Zamaroczy and Bernardi 1986; Weiller *et al.* 1989; Paquin *et al.* 2000; Smith and Lee 2008; Erpenbeck *et al.* 2009; Smith and Lee 2009; Lavrov 2010; Lang *et al.* 2014). These GC clusters have been proposed to be TE-like based primarily on their sporadic distribution (Weiller *et al.* 1989; Nakazono *et al.* 1994; Koll *et al.* 1996; Paquin *et al.* 2000; Lavrov 2010; Lang *et al.* 2014). Consistent with the hypothesis of GC clusters being TE-like, other marks of TEs including copy number variation and putative target-site duplication have been observed in GC clusters (Weiller *et al.* 1989; Lang *et al.* 2014).

Although mobility of GC clusters has been suggested in previous studies, there is a lack of compelling evidence convincingly demonstrating that GC clusters are *bona fide* mitochondrial TEs . The evolutionary fate of GC clusters is ultimately determined by their intraspecific variation and population genetics processes, and the fast growing population genomics data emerge as excellent resources for a better understanding of the nature of GC clusters. In this study, we assembled 28 complete mitochondrial genomes from *Saccharomyces cerevisiae* and *S. paradoxus*, and compared their GC clusters together with five other published *S. cerevisiae* and *S. paradoxus* mitochondrial genomes. Our results reveal that one 42-nucleotide palindromic GC cluster (GC42) is of rapid proliferation in *S. cerevisiae* and is involved in homologous-recombination–mediated genetic exchange between *S. cerevisiae* and *S. paradoxus*. GC42 and other GC clusters have highly dynamic evolutionary trajectories featuring rapid nucleotide substitutions, dynamic merger, and shuffling of GC-cluster units. Possible transposition mechanisms and evolutionary/functional consequences of GC clusters are discussed.

## Materials and Methods

### Strains and mitochondrial genome assembly

The raw Illumina sequencing reads from *S. cerevisiae* and *S. paradoxus* were obtained from the NCBI SRA database (Bergstrom *et al.* 2014). The reads were assembled using a combination of software: SOAPdenovo (Luo *et al.* 2012), SPAdes (Bankevich *et al.* 2012), Velvet (Zerbino and Birney 2008), and Consed (Gordon and Green 2013). Five different K-mers (21, 33, 55, 77, and 89) were used during the assembly processes and default settings were chosen for all remaining parameters. The best-assembled contigs were used to fill gaps using SSPACE (Boetzer *et al.* 2011) and GapFiller (Boetzer and Pirovano 2012). The assembled genomes were evaluated by mapping back the raw reads using BWA (Li and Durbin 2009). We have successfully completed mitochondrial genomes for 14 *S. cerevisiae* strains and 14 *S. paradoxus* strains (GenBank accession numbers KP712778–KP712805). The complete mitochondrial genomes of *S. cerevisiae* S288c, YJM789, YJM993, No7, and *S. paradoxus* CBS432 were obtained from the GenBank database. All 33 complete mitochondrial genomes are subject to further analysis.

### Detection and analysis of GC cluster repeats

Dispersed repeats in the *S. cerevisiae* S288c mitochondrial genome were identified using RepeatFinder implemented in the UGENE package (Okonechnikov *et al.* 2012) with a minimum length of 30 bp. Sequences with GC content more than 30% were grouped by *BLASTClust* (Altschul *et al.* 1997) with 70% length coverage and 100% sequence identity. The flanking sequences were manually inspected to define GC cluster boundaries. The four most abundant GC-rich dispersed repeats in the *S. cerevisiae* S288c genome are shown in Table 1. For convenience, the most abundant GC cluster was named after its length, *i.e.*, GC42 is a GC cluster 42 bp in length. The secondary structures of GC clusters were predicted by Mfold 4.6 with default parameters (Zuker 2003). The nucleotide variation of GC42 copies was visualized using WebLogo (Crooks *et al.* 2004). To identify homologs of GC clusters, BLASTN searches (Camacho *et al.* 2009) were performed and significant matches were required to have 90% sequence identity and 95% length coverage.

**Table 1:** The four most abundant dispersed repeats and number of identical copies in the *Saccharomyces cerevisiae* reference genome S288c

Length	Sequences	Copies	GC%
42 nt	AGTTCCGGGGCCCGGCCACGGGAGCCGGAACCCCGAAAGGAG	25	75
38 nt	ACTCCTTCGGGGTCCGCCCCGCGGGGGCGGGCCGGACT	7	85.7
32 nt	ACTCCTTCGGGGTCCCCGCCGGGGCGGGGACT	6	82.8
42 nt*^a^*	AGTTCCGGGGCCCGGCCACGGGAGCCGGAACCCCG**G**AAGGAG	5	77.5

a The fourth most abundant repeat differs by a single nucleotide (position 36 and in bold) from the top repeat sequence.

### Phylogenetic and phylogenomic analysis

After mining the nuclear genomic data (Liti *et al.* 2009), 630 nuclear-encoded single-copy genes were universally present in all 18 *S. cerevisiae* and 15 *S. paradoxus* strains, and all 630 genes were included in further phylogenomic analysis. Each gene was aligned individually using MUSCLE (Edgar 2004). The concatenated sequences of all gene alignments were used to reconstruct the phylogenetic relationship of these strains. Phylogenetic trees were constructed using the RAxML program (Stamatakis 2006) under a GTR + Γ substitution model, and 100 bootstrap iterations were performed. The phylogenetic relationship was constructed for homologous flanking regions using 100 nt upstream and 100 nt downstream of the target GC42 homologous position. In *S. paradoxus*, the same homologous position can have standing-alone GC42 in some strains and merged GC cluster in other strains. We have manually inspected the sequence alignment of the homologous flanking ±100-bp regions.

### Transcriptome data analysis and GC42 expression

The pair-ended (100PE) raw RNA-seq data of two strains, GCDA5 and GCDA8, isogenic to wild-type *S. cerevisiae* S288c (Turk *et al.* 2013) were obtained from the NCBI SRA database (SRR900186 and SRR900220 for GCDA8; SRR900222 and SRR900223 for GCDA5). This dataset is ideal for the examination of GC42 expression, because the sequencing libraries were prepared with no DSN treatment, no polyA selection, no ribosomal or tRNA subtraction, and no size selection. This study is not concerned about exon/intron junctions that are only present in the *cox1*, *cob*, and 21S rRNA genes, and BWA (Li and Durbin 2009) has been shown to be superior to other mapping programs on RNA-seq reads 100 bp in length (Lindner and Friedel 2012). We chose BWA with default settings to directly map RNA-seq reads onto the reference mitochondrial genome. The expression levels for individual GC42 copies and intron-lacking protein coding genes were calculated as RPKM with Artemis v16.0.0 (Carver *et al.* 2012). The RPKMs were normalized to have identical *atp6* RPKMs (*i.e.*, $\sqrt{xy}$, *x* is the *atp6* RPKM in GCDA8, *y* is the *atp6* RPKM in GCDA5) between the GCDA5 and GCDA8 strains.

### Quantification of the GC42 turnover rates

The distribution of GC42 was mapped on the phylogenetic tree. Gain and loss at homologous sites were modeled as a two-state continuous-time Markov process, with states 0 (absence) and 1 (presence) on a phylogeny using the tree branch length as a relative time scale in the R package DiscML (Kim and Hao 2014). The turnover rate is expressed as the number of gains/losses per site per nucleotide substitution (Hao and Golding 2006; Wu and Hao 2014). The GC42 turnover rates were estimated using the simplistic (one-parameter) model by constraining the gain and loss rates to be the same and the two-parameter model separating the gain and loss rates. The ancestral state for each GC42 homologous position was estimated using BayesTraits (Pagel *et al.* 2004).

The polymorphic level of GC42 presence/absence was compared against those in five nuclear-encoded transposons (Ty1–Ty5) in *S. cerevisiae*. The phylogenetic distribution of each Ty transposon was obtained from (Carr *et al.* 2012). The pairwise difference was calculated as $\frac{\#\;different\;}{\#\;identical\;+\;\#\;different\;}$; sites with missing information were excluded from each pairwise comparison. The choice of performing pairwise comparison was made because the Ty presence/absence data, unlike the GC42 distribution data, contained missing information for most homologous positions and were unsuitable for reliable turnover rate estimation.

## Results

### Rapid turnover of GC42

TEs are often of a high copy number in the genome. We initiated a search for dispersed repeats with high GC content in the reference *S. cerevisiae* S288c mitochondrial genome. The four most abundant GC clusters range from 5 to 25 identical copies in the *S. cerevisiae* S288c mitochondrial genome (Table 1). These identified GC clusters are all flanked by short direct repeats (AG or ACT) in a manner similar to target-site duplication in class II TEs (DNA transposons). To obtain a detailed picture of the mobility of GC clusters, the most redundant 42-nucleotide GC cluster (GC42) was chosen for further comprehensive analysis. Given the fact that TEs are subject to degeneration (Carr *et al.* 2012; Bleykasten-Grosshans *et al.* 2013), the search criterion for GC-cluster homologs was relaxed to 90% sequence identity and 95% match in length to the query (see *Materials and Methods*).

**Figure 1:**
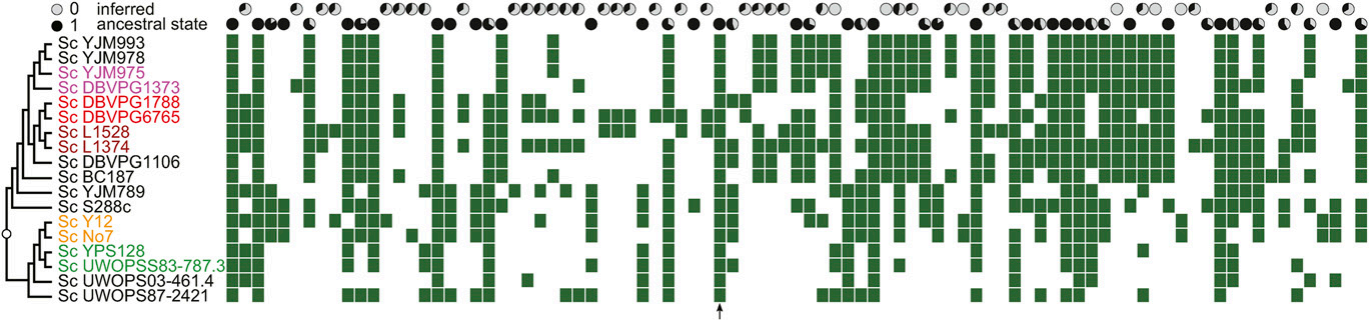
Distribution of GC42 homologs among 18 *S. cerevisiae* strains. For each homologous position, the likely ancestral state was estimated and shown in a pie chart. The pie charts on the top row favor an ancestral state of absence, whereas the pie charts on the second row favor an ancestral state of presence. Five closely related pairs of genomes were subject to detailed analysis of GC42 density in Figure 2; each pair is shown in a distinct color. The arrow refers to a GC42 homologous position that is shared between *S. cerevisiae* and *S. paradoxus* and involved in phylogenetic analysis for horizontal transfer in Figure 4.

In *S. cerevisiae*, we identified 89 GC42 positions, with five present in the *cob* introns, two in the *cox1* introns, and the remaining 82 GC42 positions at intergenic regions. Consistent with previous studies (Weiller *et al.* 1989), GC42 is sporadically distributed among the conspecific strains in *S. cerevisiae* (Figure 1). To further demonstrate whether the sporadic GC42 distribution resulted from multiple independent losses or from GC42’s own mobility, we sought a quantitative approach to measure the rates of GC42 gain and loss. If the sporadic GC42 distribution resulted from multiple independent losses, one should expect a negligibly low rate of GC42 gain but a substantially high rate of GC42 loss. If the sporadic GC42 distribution resulted from GC42’s own mobility, both the rates of GC42 gain and loss are expected to be high. The overall rate (± SE) of GC42 gain in *S. cerevisiae* was estimated to be 135.2 ± 8.0 gains per site per nucleotide substitution (Table 2, see *Materials and Methods* for detailed explanation). That is, that GC42 gain takes place at a rate approximately two orders of magnitude higher than nucleotide substitution. The rate of GC42 loss was estimated to be 235.2 ± 14.0 (Table 2). The high rates of GC42 turnover support the hypothesis that GC42 is of high mobility. Furthermore, GC42 appears to be more presence/absence polymorphic than all five nuclear-encoded Ty transposons (Ty1–Ty5) in *S. cerevisiae* (Supporting Information, Figure S1), which is consistent with high GC42 mobility. Unfortunately, reliable turnover rate estimation could not be performed on the Ty data, because they contain missing information at most identified Ty positions due to the relatively low sequence coverage in the nuclear genomes.

**Table 2:** GC42 turnover rates (± SE) estimated for different phylogenetic groups in *S. cerevisiae* and *S. paradoxus*

	One Rate Parameter		Two Rate Parameters			
Phylogenetic Groups	Rate (μ)	Ln*L*	Gain	Loss	Ln*L*	2ΔLn*L*
*S. cerevisiae*	165.1 ± 9.8	−749.2	135.2 ± 8.0	235.2 ± 14.0	−742.5	13.4[Table-fn t2n1]
Clade A*^a^* in *S. cerevisiae*	327.4 ± 26.2	−415.6	354.9 ± 28.4	289.1 ± 23.1	−415.1	1.0
*S. paradoxus*	489.3 ± 64.9	−150.0	274.1 ± 34.3	808.3 ± 101.0	−138.9	22.2[Table-fn t2n1]
Clade B*^a^* in *S. paradoxus*	977.9 ± 152.2	−97.5	1236.8 ± 201.3	898.8 ± 146.3	−97.1	0.8

a The clades are per Figure S1.

A notable bias toward GC42 loss was observed in *S. cerevisiae* (Table 2), which can be explained by the deleterious nature of GC42 as a type of TEs and perhaps transient fate at many mitochondrial genomic locations. Under such circumstances, one would expect higher GC42 turnover rates and less bias toward loss among more closely related genomes due to recent evolutionary separation, which does not yet provide sufficient time to purge deleterious genetic elements. Consistently, when estimation was performed within more closely related clades (*i.e.*, clade A and clade B in Figure S2), the turnover rates are higher than those estimated for the entire species and there is an insignificant trend toward GC42 gain, at minimum, and no more bias toward GC42 loss (Table 2). Furthermore, the ancestral state at each GC42 homologous position was estimated (Figure 1). Among the 89 GC42 positions, 46 positions favor ancestral “presence” and 43 positions favor ancestral “absence.” The ancestral absence of GC42 in these positions supports a significant number of GC42 gains during mitochondrial genome evolution. Thus, the sporadic distribution of GC42 homologs is likely due to their own dynamic lifecycle as TEs.

### GC42 is under functional constraint

**Figure 2:**
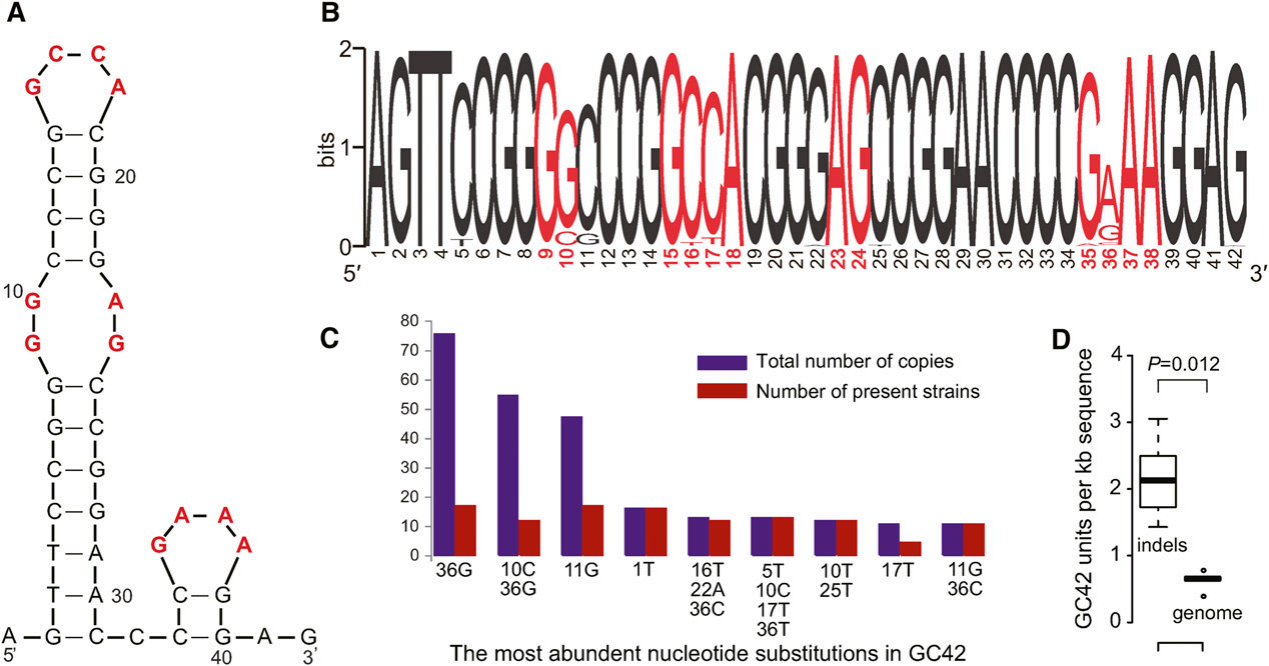
Characteristics of GC42 sequences in *S. cerevisiae*. (A) Predicted secondary structure of GC42 based on the consensus sequence. The nucleotides in loop regions are in red. (B) Sequence logo for all GC42 homologous sequences. The nucleotides in loop regions are in red. (C) Distribution of the nine most abundant GC42 sequence types. For each GC42 sequence type, the total GC42 copy number in all *S. cerevisiae* strains and the number of strains containing the corresponding sequence type are shown. (D) GC42 density (units/kb) in indel regions compared with that in whole genomes in the five pairs of closely related genomes. The *P*-value is based on the Mann–Whitney *U*-test.

TEs can move from one genomic location to another faster than the genome can replicate. The reproductive success of TEs will depend on their ability to rapidly proliferate within the genome. To maintain their functional integrity, the TE sequences are expected to be under selection to purge mutations disrupting TE activity. GC clusters are known to form palindromic structures (Figure 2A) (de Zamaroczy and Bernardi 1986; Lang *et al.* 2014), and we sought whether their palindromic structures are under functional constraint. If GC42 is under no functional constraint, then it would be subject to random substitutions along the 42 nucleotides. Among all the GC42 homologs in *S. cerevisiae*, 367 nucleotide changes (by comparing to the consensus sequence in Figure 2B) are located at 12 sites in the loops regions, whereas 174 nucleotide changes are at the remaining 30 nucleotide sites (*P* = 8.04×10^−7^, Fisher’s exact test). The significantly high proportion of nucleotide changes in the loop regions suggests that the secondary hairpin structure of GC42 is of functional importance. The two most abundant GC42 homologs distinct from the consensus (36^G^, 10^C^36^G^) only have nucleotide changes in the loop regions (Figure 2C). These substitution-containing homologs of GC42 also show copy number variation among strains following a sporadic distribution (Figure 2C). The constraint on the secondary structure suggests that the palindromic structure is functionally important for GC42.

The average nucleotide diversity was measured separately for the loop and stem regions of GC42, and was compared against the average pairwise synonymous substitution rates in seven (all but the *var* gene) protein genes (Figure S3). Here, we used the average pairwise synonymous substitution rates as an approximate guide for genome-wide mutation rate due to the difficulty to accurately align the entire sequences of extremely AT-rich mitochondrial genomes. The average nucleotide diversity of the GC42 loop regions is higher than the median pairwise synonymous substitution rates of most protein coding genes. This could be explained by nonallelic homologous recombination among the GC42 sequences, by purifying selection acting on gene synonymous sites (Lawrie *et al.* 2013), and/or by targeted copy correction by gene conversion on protein coding genes (Khakhlova and Bock 2006; Christensen 2013). Importantly, the diversity of the GC42 stem regions is much lower than the pairwise synonymous substitution rates of most protein coding genes. Conservatively speaking, the substitution rate in the GC42 stem regions has been significantly reduced from the genome-wide mutation rate, thus GC42 is believed to be under functional constraint.

Sequence transposition in one genome leads to insertions/deletions (indels) in a two-genome comparison. Recent TE activity can generate genomic indels, which in turn serve as indicators for recent TE activity (Mills *et al.* 2007). In Figure 1, some closely related genomes show very different GC42 distribution, suggesting that GC42 has recently been, and/or still is, active. In five pairs of closely related genomes (colored in Figure S2), we have identified sequences (indels) present in one genome but not the other, and found that the density of GC42 (units/kb) is significantly higher in these indels than in the whole genomes (Figure 2D). The high GC42 density in the indels of closely related genome pairs is likely contributed by the recent TE activity of GC42.

**Figure 3:**
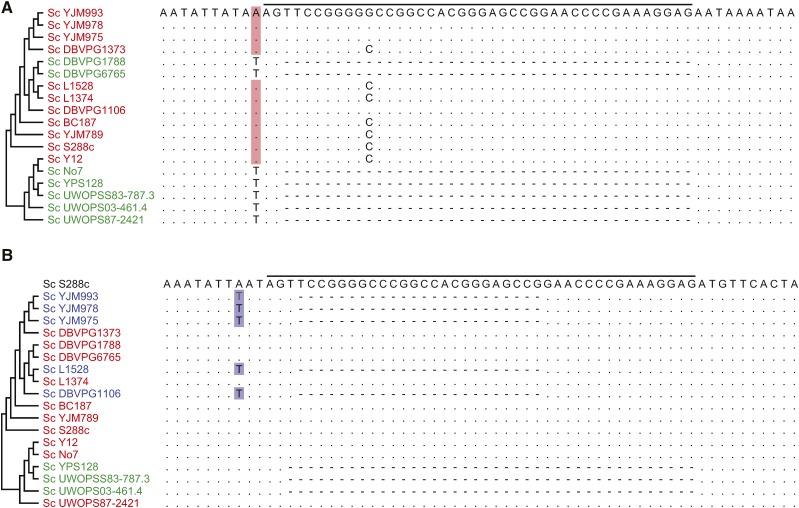
Nucleotide alignment of two GC42 homologous positions and their flanking regions. (A) GC42 presence and absence. (B) GC42 presence, GC42 subunit presence, and GC42 absence. The GC42 unit is overlined. Dots indicate identities relative to the *top* sequence in each panel, whereas letters represent nucleotide differences. The strain names are colored by their GC42 pattern: GC42 presence in red, GC42 subunit presence in blue, and GC42 absence in green. The putative co-conversion tract nucleotides are colored for GC42 presence or GC42 subunit presence in the same color with the corresponding strain.

Nucleotide substitutions immediately flanking GC42 were observed (Figure 3). Importantly, the variation in these nucleotide sites is associated with the presence or absence of GC42, but not necessarily always with the phylogenetic relationship. These findings suggest that the variable nucleotides immediately flanking GC42 are likely the co-conversion tract of GC42 insertion, a common sequence mark of insertion for many mobile sequences, *e.g.*, group I and group II introns (Lambowitz and Belfort 1993; Moran *et al.* 1995; Sanchez-Puerta *et al.* 2008). The presence of putative GC42 co-conversion tract suggests active mobility of GC42 at some point of evolution.

### Exchange of GC42 between species

**Figure 4:**
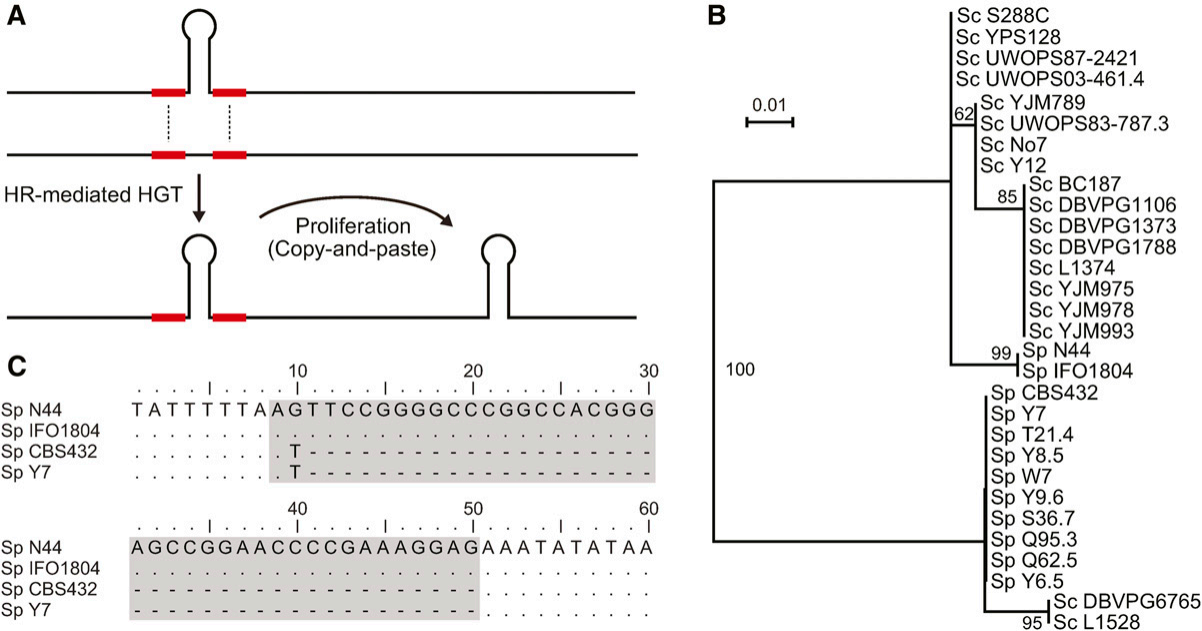
GC42 exchange between *S. cerevisiae* and *S. paradoxus*. (A) Schematic presentation of horizontal transfer and propagation into new host. (B) Evidence of horizontal transfer. Phylogenetic analysis for the flanking ±100-bp homologous regions of a GC42 unit (arrow in Figure 1). (C) Evidence for proliferation after horizontal transfer. Sequence alignment of the homologous region for the second GC42 in N44 and IFO1804 strains. The GC42 position is highlighted in gray. Dots indicate identities relative to the *N44* GC42 sequence, whereas letters represent nucleotide differences.

Many TE families are known to have horizontal transmission for their long-term maintenance during evolution (Schaack *et al.* 2010; Wallau *et al.* 2012). To explore whether GC42 is involved in horizontal transfer and proliferation in other species (Figure 4A and Figure S2), we searched GC42 homologs in 15 *S. paradoxus* strains. GC42 homologs in two *S. paradoxus* strains (N44 and IFO1804) show 100% identity with the GC42 at the homologous position in *S. cerevisiae* S288c (also the most abundant GC42 type shown in Table 1), and the flanking regions in these two *S. paradoxus* strains show much closer relationships with *S. cerevisiae* than any other *S. paradoxus* strains (Figure 4B). These suggest that the transfer of GC42 into *S. paradoxus* N44 and IFO1804 from *S. cerevisiae* was mediated by homologous recombination. Moreover, *S. paradoxus* N44 and IFO1804 bear a second GC42 homolog of 100% identity with the first GC42 copy. In a BLASTN search using the flanking region of the second GC42 as a query, significant hits were found in only two other *S. paradoxus* strains, CBS432 and Y7; however, both strains lack GC42 at this homologous position (Figure 4C). Because no other GC42 homologs in *S. paradoxus* than these two copies in N44 and IFO180 are 100% identical with any GC42 homologs in *S. cerevisiae*, it is thus most likely that the second GC42 copy in *S. paradoxus* N44 and IFO1804 was inserted recently and perhaps due to proliferation after the transfer of the first GC42 from *S. cerevisiae*.

**Figure 5:**
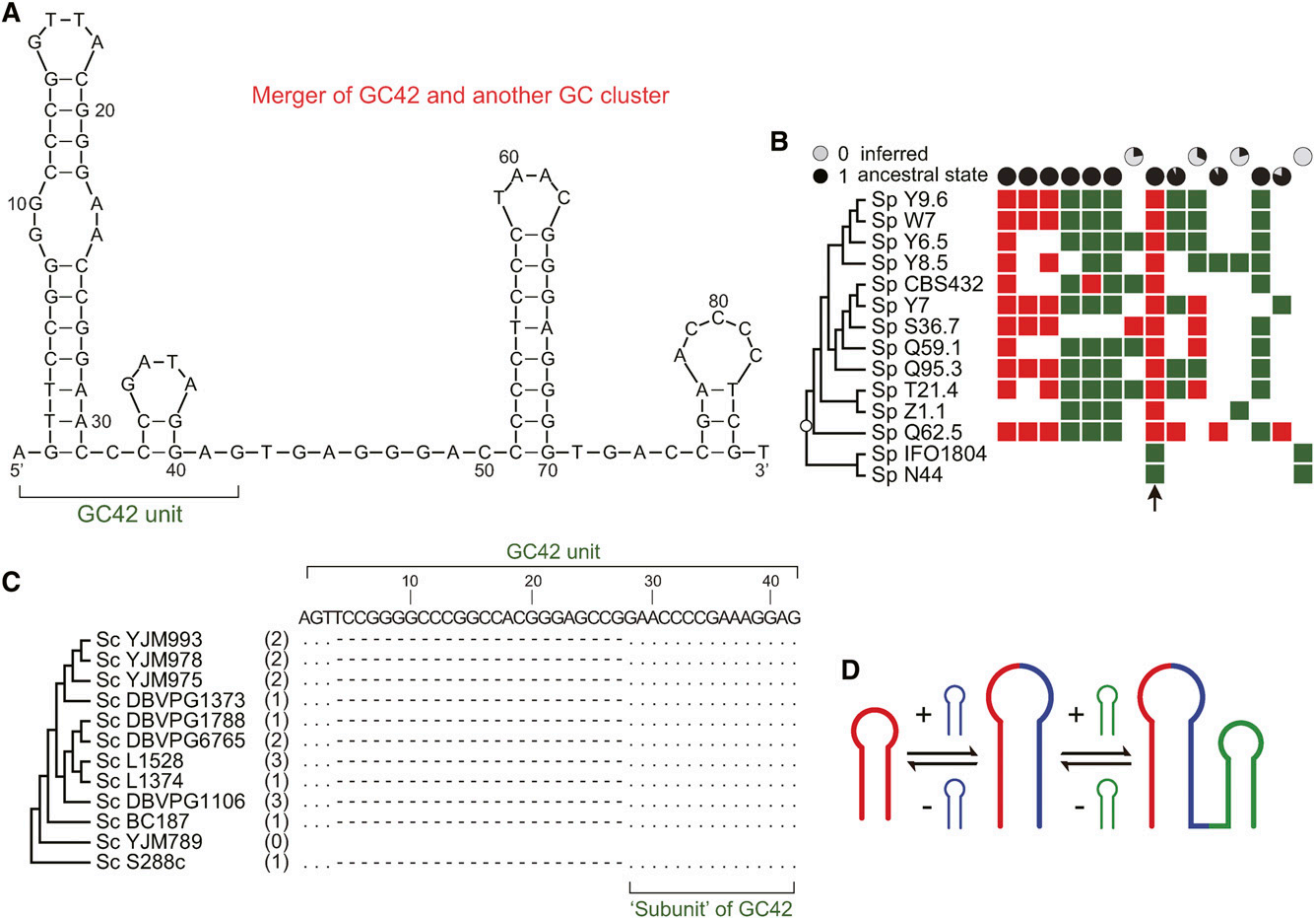
Merger and shuffling of GC42. There are two types of GC42 in *S. paradoxus*: stand-alone and part of a merged 86-nucleotide GC cluster. (A) Predicted secondary structure of the merged 86-nucleotide GC cluster in *S. paradoxus*. (B) Distribution of stand-alone GC42 (green) and merged GC42 (red) in *S. paradoxus*. For each homologous position, the likely ancestral state was estimated and shown in a pie chart. The pie charts on the top row favor an ancestral state of absence, whereas the pie charts on the second row favor an ancestral state of presence. (C) Multiple alignment of GC42 and their precursor GC18 in *S. cerevisiae*. (D) Schematic presentation of merger and shuffling of GC clusters.

It is also evident that the GC42 flanking regions in two *S. cerevisiae* strains (L1528 and DBVPG6765) are more closely related to *S. paradoxus* than any other *S. cerevisiae* strains (Figure 4B). The GC42 homologs in *S. cerevisiae* L1528 and DBVPG6765 share higher similarity with the consensus GC42 sequence in *S. paradoxus* (98% identity) than that in *S. cerevisiae* (88.1% identity). These results suggest that GC42 exchange also takes place from *S. paradoxus* to *S. cerevisiae* via homologous recombination at the flanking regions. Similarly, GC42 is sporadically distributed in *S. paradoxus* strains, ranging from 2 to 11 copies. GC42 homologs in *S. paradoxus* also show a higher proportion of nucleotide changes in the loop regions than in the stem regions. Among the 113 GC42 homologs in *S. paradoxus*, 48 nucleotide changes are located at 12 sites in the loop regions, whereas 2 nucleotide changes are at the remaining 30 nucleotide sites (*P* = 2.42×10^−12^, Fisher’s exact test). It is worth noting that the ratio of nucleotide changes in the loops over in the stems (48:2) in *S. paradoxus* is higher than that (367:174) in *S. cerevisiae* (*P* = 3.27×10^−06^, Fisher’s exact test). These results could be explained by the greater diversity in loop regions among the *S. paradoxus* strains than that among the *S. cerevisiae*, and a higher fraction of nucleotide changes in the stems have been purged in *S. paradoxus* than in *S. cerevisiae*. Despite its relative low copy number in *S. paradoxus*, GC42 should still be considered mobile in *S. paradoxus*. In fact, the estimated turnover rates in *S. paradoxus* and in clade B are higher than those in *S. cerevisiae* and in clade A (Table 2). Among the 15 GC42 homologous positions in *S. paradoxus*, four positions were inferred to favor an ancestral state “absence” (Figure 5). Furthermore, GC42 exchange can take place at homologous positions between different genomes, which would not be detectable in the analysis of turnover rates.

### GC cluster merger and the birth of new GC clusters

**Figure 6:**
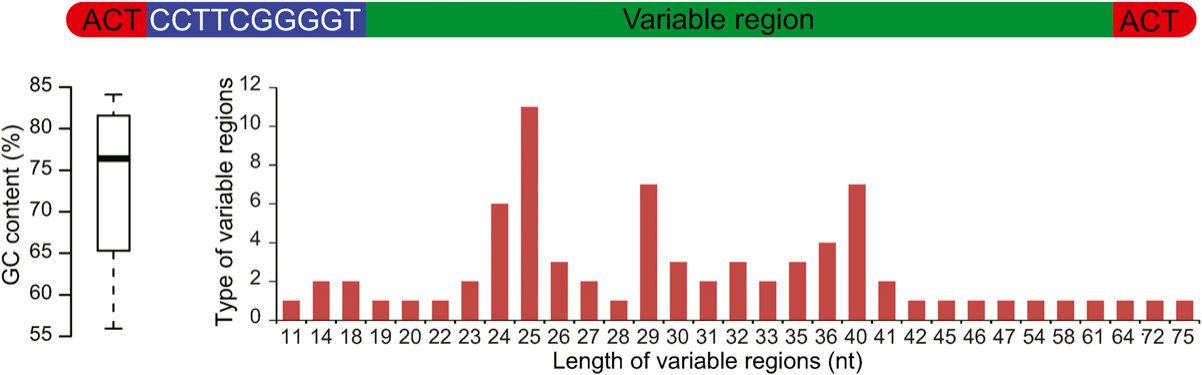
Distribution of GC clusters that share an identical 13-nucleotide sequence at the 5′-end. The boxplot refers to GC content for all GC clusters sharing GC13 (conserved region). The a-axis indicates the length of GC-rich sequences adjacent to the identical 13-nucleotide sequence, whereas the y-axis indicates the number of unique sequence types for each sequence length.

In *S. paradoxus*, GC42 can be found in two forms, stand-alone and merged with another GC cluster (Figure 5A), both of which show copy number variation among different strains (Figure 5B). The stand-alone and merger forms can be found in different strains at the same homologous site, determined by their homologous flanking regions (Figure 5B). We then sought to address whether the merger of GC clusters is a general phenomenon during the evolution of GC clusters. One 18-nucleotide GC cluster immediately flanked by AT-rich sequences was found to be identical with the 3′-terminus of GC42 (Figure 5C). This GC18 sequence was found in 11 of the 18 studied *S. cerevisiae* strains and of copy number variation, but was absent from any *S. paradoxus* strains (Figure 5C). Like intact GC42, GC18 also has putative co-conversion tracts. In one GC42 homologous position (Figure 3), all the T nucleotide substitutions are associated with the presence of GC18. These results suggest that GC18 might be a smaller mobile unit than GC42 and raise the possibility that GC42 itself could have resulted from merger of two smaller GC clusters (Figure 5D). We further noticed that the second and third most abundant GC clusters in *S. cerevisiae* S288c share an identical 5′-terminus (Table 1). Using the 5′-terminus as a query, we have identified 403 GC-rich sequences in the 18 studied *S. cerevisiae* strains, all of which share the first 13 nucleotides 5-ACTCCTTCGGGGT-3 but might have different downstream adjacent sequences (Figure 6). These sequences are of 76 distinct sequence types with various lengths. Thus, it seems to be common that GC clusters undergo active merger and shuffling during yeast mitochondrial genome evolution.

### Expression of the GC42 sequences

**Figure 7:**
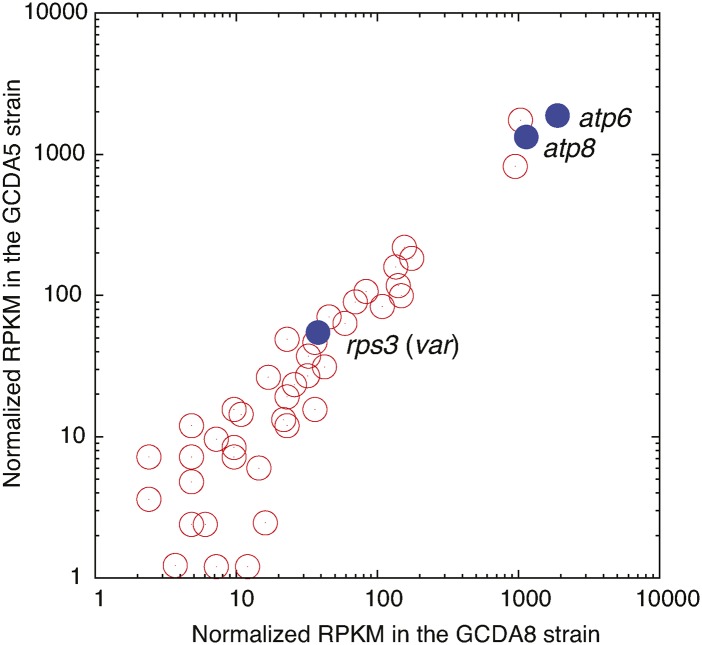
Expression of GC42 homologs. As a comparison, three protein genes, *rps3* (*var*), *atp8*, and *atp6*, are shown. The RPKMs are normalized to have identical *atp6* RPKMs between the GCDA5 and GCDA8 strains, both of which are derived from *S. cerevisiae* BY4741 (isogenic to S288c).

Many GC42 sequences are transcribed into RNAs, but their express levels vary substantially (Figure 7). Twelve GC42 homologs show higher expression levels than the var (rps3) gene, and two GC42 homologs show high expression levels comparable to *atp8* and *atp6*. We further investigated the expression level of the 12 most highly expressed GC42 sequences and their flanking sequences (upstream 40 nucleotides and downstream 40 nucleotides). We have observed that seven GC42 sequences are transcribed at levels similar to their flanking sequences (*e.g.*, in Figure S4, A, D, E, G, H, J, and K) and five GC42 sequences are transcribed at higher levels than their flanking sequences (*e.g.*, in Figure S4 B, C, F, I, and L). These results suggest that at least some GC42 sequences are independently transcribed in the host.

## Discussion

### The origin and evolutionary history of GC-rich TEs

During evolution, the GC-rich TEs both accumulate nucleotide substitutions and undergo unit merging and shuffling. Nucleotide changes in *S. cerevisiae* and *S. paradoxus* strains all support a higher proportion of nucleotide changes in the loop regions than in the stem regions. This is also supported by the interspecific difference of GC42 homologs between *S. cerevisiae* and *S. paradoxus*. For instance, the GC42 consensus sequence in *S. cerevisiae* (Figure 2A) differs by four nucleotides (positions 16, 17, 24, 37) from that in *S. paradoxus* (Figure S5) and all four nucleotides are in the loop regions (*P* = 0.011, Fisher’s exact test). GC42 undergoes dynamic transformation, presumably derived from merger of smaller GC clusters as observed in *S. cerevisiae* and further merging into bigger GC clusters as observed in *S. paradoxus* (Figure 5). GC clusters can potentially undergo merger and separation (fusion and fission) in a bidirectional manner, but the abundance of each GC cluster unit depends on its own mobile activity and functional constraint under selection.

**Figure 8:**
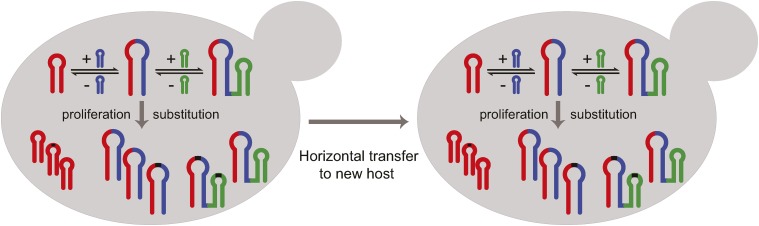
Model for the lifecycle of GC-rich TEs. GC-rich TEs undergo rapid substitution, dynamic merger, shuffling, and horizontal transfer, with details discussed in the main text.

Our study observed exchange of GC42 between *S. cerevisiae* and *S. paradoxus* via homologous recombination at the conserved flanking regions, which was recently recognized as an often overlooked mechanism mediating horizontal transfer (Polz *et al.* 2013). Similarly, mitochondrial introns have been previously documented to be involved in horizontal transfer mediated by homologous recombination at the conserved flanking regions (Hepburn *et al.* 2012; Wu and Hao 2014). Horizontal transfer of another GC cluster has been documented, which is involved in the transfer of a 48-nucleotide GC cluster within the var (rps3) gene between *S. cerevisiae* and *Kluyveromyces lactis* (Lang *et al.* 2014). Given the similar dynamics of GC clusters observed in *S. cerevisiae* and *S. paradoxus*, we propose that after horizontal transfer of GC-rich TEs, these TEs would experience the similar evolutionary dynamics (nucleotide substitutions, merging, and shuffling) as in the previous host (illustrated in Figure 8). This process shares all the important features documented in animal TEs, *e.g.*, rapid mutation accumulation, diversification, and proliferation in a host and possible horizontal transfer into a new host (Schaack *et al.* 2010).

### Amplification mechanism of GC-rich TEs

GC clusters, including GC42, bear putative target-site duplication (Weiller *et al.* 1989; Lang *et al.* 2014) (Figure 2B and Table 1), a key feature in class II TEs (DNA transposons). It has been proposed that endonucleases encoded in mitochondrial introns may facilitate the mobility of GC clusters as DNA transposons (Lang *et al.* 2014). However, target-site duplication in class II TEs is involved in a cut-and-paste transposition mechanism, which does not often lead to a substantial increase in copy numbers. There is one possibility for a cut-and-paste transposition mechanism to increase copy numbers. It would require the combination of stable heteroplasmic mtDNAs containing different GC42 patterns, relatively efficient mtDNA recombination and segregation, and biased retention of high GC42-copy mtDNA genotypes. Among these three requisites, only mtDNA recombination is commonly recognized (Shannon *et al.* 1972; Dujon *et al.* 1974; Fritsch *et al.* 2014). The heteroplasmic state in *S. cerevisiae* is generally believed to be transient and to last no more than 20 mitotic cell divisions (vegetative segregation) (Birky 2001), and there has been no evidence supporting GC cluster–rich mtDNA genotypes being preferentially retained from heteroplasmic cells. A similar challenge has been documented in a previous study on the copy number variation of nuclear MITEs (miniature inverted-repeat transposable elements) (Fattash *et al.* 2013). To obtain a more sophisticated answer on the transposition and proliferation of GC-rich TEs, we sought to access the possible contribution of the cut-and-paste mechanism in GC-rich TEs. If cut-and-paste were the only transposition mechanism of GC42, then we would expect all GC42-absence positions to have either one single set of target site nucleotides (AG) (presumably never inserted or perfectly cut out) or GC42 fragment with two sets of target site nucleotides (AG) as footprint. Among the 89 homologous positions, GC42-absence sites in 25 homologous positions only have a single set of target site nucleotides, and five positions have footprint. All these findings suggest that cut-and-paste is unlikely the sole mechanism of GC42 transposition.

We then investigated the possibility of GC-rich TEs being involved in RNA-mediated transposition (also known as copy-and-paste). The expression of GC42 would be an excellent precondition for reverse transcription (Figure 7). Furthermore, yeast mitochondrial genomes contain several functional intron ORFs, which encode endonucleases and reverse transcriptases involved in intron mobility (Eskes *et al.* 2000; Lang *et al.* 2014). The presence of functional reverse transcriptase, in principle, can facilitate RNA-mediated retrotransposition of GC-rich TEs. We suspect that the rapid GC42 proliferation is, in part, due to RNA-mediated retrotransposition (copy-and-paste). However, additional experimental evidence is required to conclusively determine the proliferation mechanisms. Previous studies of mitochondrial intron transposition and retrotransposition have documented the co-conversion of flanking exon sequences as the result of mobile intron insertion mediated by intron-encoded enzymes (Lambowitz and Belfort 1993; Moran *et al.* 1995; Sanchez-Puerta *et al.* 2008). The putative co-conversion tracts are likely the footprints of GC cluster insertions (Figure 3).

### Merger and shuffling of palindromic clusters: a potential source of evolutionary novelty

Palindromic GC clusters were first discovered approximately 40 years ago (Bernardi 1976), but the origin and evolution of GC clusters are still poorly understood. Our findings show, for the first time, that GC clusters undergo dynamic merger and shuffling (Figure 5). It is reasonable to believe that GC clusters have variable mobile activities because of their different secondary structures. The highly dynamic merger and shuffling processes will alter the secondary structure and mobile activity of GC-rich TEs and ultimately change their abundance at both the genomic and population levels.

The merger of palindromic sequences has been proposed as an important mechanism to create functional and structurally complex RNAs. For instance, nuclear tRNA halves can form hairpin structure and can be ligated into chimeric tRNA with cloverleaf structure during evolution (Zuo *et al.* 2013), that is, merger and shuffling of tRNA fragments created modern tRNAs. Modern tRNAs could have been inserted into the genome via retrotransposition (Zuo *et al.* 2013), which is likely also crucial for the mobility of GC clusters. Many hairpin-structured RNAs have been previously shown to bear ribozyme activity, which catalyzes self-cleavage and ligation reactions (Gwiazda *et al.* 2012; Muller *et al.* 2012). The potential ribozyme activity of palindromic sequences could play an important role in initiating and promoting their own merger and shuffling. We tend to believe that the shuffling of hairpin-forming sequences is likely associated with RNA-mediated ligation and retrotransposition.

### Evolution of GC clusters and mitochondrial genome size

Most GC clusters, including GC42, are located in intergenic regions, whose size is often strongly associated with mitochondrial genome size (Bouchier *et al.* 2009). GC clusters are short in length, and thus their direct sequence-length contribution to mitochondrial genome size is minimal. However, GC clusters have been suggested as mtDNA recombination hotspots (Dieckmann and Gandy 1987), and GC cluster–mediated gene conversion can insert or delete large genomic fragments, which ultimately lead to substantial alteration of genome size. GC clusters have also been proposed to play a role in increasing mitochondrial genome size by inducing long AT-rich stretches (Bouchier *et al.* 2009). Furthermore, the abundance of TE sequences has been shown as the result of nonadaptive processes such as mutation and genetic drift during the evolution of genome size (Lynch and Conery 2003; Lynch *et al.* 2006). The nonadaptive evolutionary theory (Lynch and Conery 2003; Lynch *et al.* 2006) would predict GC clusters, as one type of TE sequence, to be more abundant in mitochondrial genomes under stronger genetic drift. Our findings suggest that the merger and shuffling processes can change the mobile activity of GC clusters, which will determine the abundance of GC clusters and ultimately influence mitochondrial genome size evolution. As more abundant population genomics data become available from a broad spectrum of species, the above hypotheses can be tested in a more rigorous and sophisticated manner.

## Supplementary Material

Supporting Information
