# Supplementary material for: A Dynamic Mobile DNA Family in the Yeast Mitochondrial Genome
Source: G3 (Bethesda). 2015 Apr 20;5(6):1273–82. doi: 10.1534/g3.115.017822 (PMC4478555; doi:10.1534/g3.115.017822)
Supplement: Supporting Information [file supp_5_6_1273__index.html]

A Dynamic Mobile DNA Family in the Yeast Mitochondrial Genome — Supporting Information 

# A Dynamic Mobile DNA Family in the Yeast Mitochondrial Genome

## Supporting Information for Wu and Hao, 2015

**Files in this Data Supplement:**

- Supporting Information - Figures S1-S5 (PDF, 261 KB)
- Figure S1 - High presence/absence polymorphism in mitochondrial-encoded GC42 in comparison of five nuclear-encoded transposons (Ty1-Ty5) in *S. cerevisiae*. (PDF, 140 KB)
- Figure S2 - Maximum likelihood phylogeny reconstructed using concatenated sequences of 630 aligned single-copy genes that are universally present in all *S. cerevisiae* and *S. paradoxus* strains. (PDF, 137 KB)
- Figure S3 - Boxplots of pairwise synonymous nucleotide diversity in seven mitochondrial protein-coding genes, and the average pairwise nucleotide diversity in the GC42 loop region (shown as red lines) as well as the GC42 stem region (shown as blue lines) from *S. cerevisiae* (Sc) and *S. paradoxus* (Sp). (PDF, 136 KB)
- Figure S4 - Expression levels of the 12 most highly expressed GC42 sequences and their (40-nucleotide upstream and 40-nucleotide downstream) flanking sequences. (PDF, 195 KB)
- Figure S5 - Characteristics of GC42 sequences in *S. paradoxus*. (PDF, 148 KB)
