# Supplementary material for: A Dynamic Mobile DNA Family in the Yeast Mitochondrial Genome
Source: G3 (Bethesda). 2015 Apr 20;5(6):1273–82. doi: 10.1534/g3.115.017822 (PMC4478555; doi:10.1534/g3.115.017822)
Supplement: Supporting Information [file supp_g3.115.017822_017822SI.pdf]

G3 · Genes | Genomes | Genetics

A dynamic mobile DNA family in the yeast mitochondrial genome

Baojun Wu and Weilong Hao*

Department of Biological Sciences, Wayne State University, Detroit, Michigan, USA

*Corresponding author: Department of Biological Sciences, 5047 Gullen Mall, Wayne State University, Detroit, MI 48202, Phone (313) 577-6450, Fax (313) 577-6891, Email: haow@wayne.edu

Data deposition: GenBank accession numbers KP712778-KP712805

DOI: 10.1534/g3.115.017822

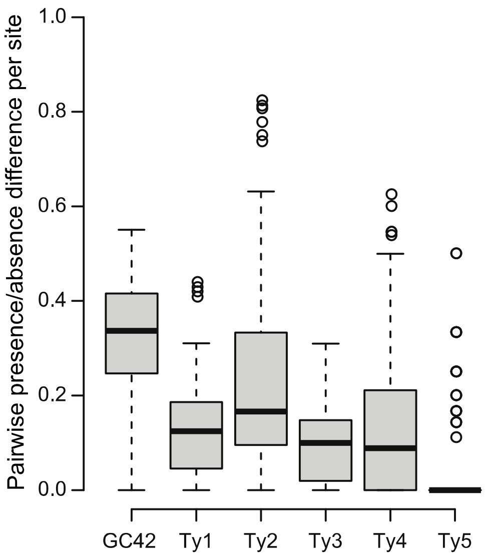

Figure S1 High presence/absence polymorphism in mitochondrial-encoded GC42 in comparison of five nuclear-encoded transposons (Ty1-Ty5) in *S. cerevisiae.* The presence/absence pattern of Ty transposons is obtained from Carr et al. (2012). The pairwise difference is calculated as $\frac{\#\, different}{\#\, identical + \#\, different}$, sites with missing information are excluded from each pairwise comparison.

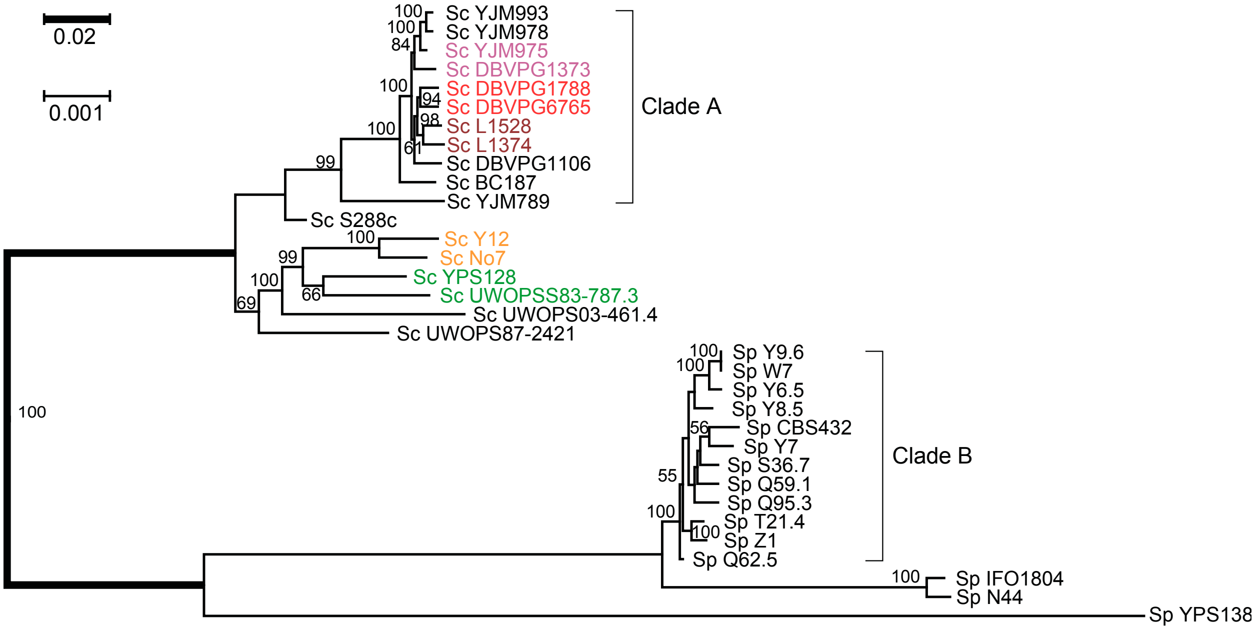

Figure S2 Maximum likelihood phylogeny reconstructed using concatenated sequences of 630 aligned single-copy genes that are universally present in all *S. cerevisiae* and *S. paradoxus* strains. Two closely related clades (clade A and clade B) are subject to further analysis on GC42 turnover (Table 1). Five closely related pairs of genomes were subject to analysis of GC42 density in the indel regions, each pair is shown in a distinct color. YJM975 was chosen to compare against DBPG1373, since no indels >30nt were observed among the mitochondrial genomes of YJM975, YJM993, and YJM978.

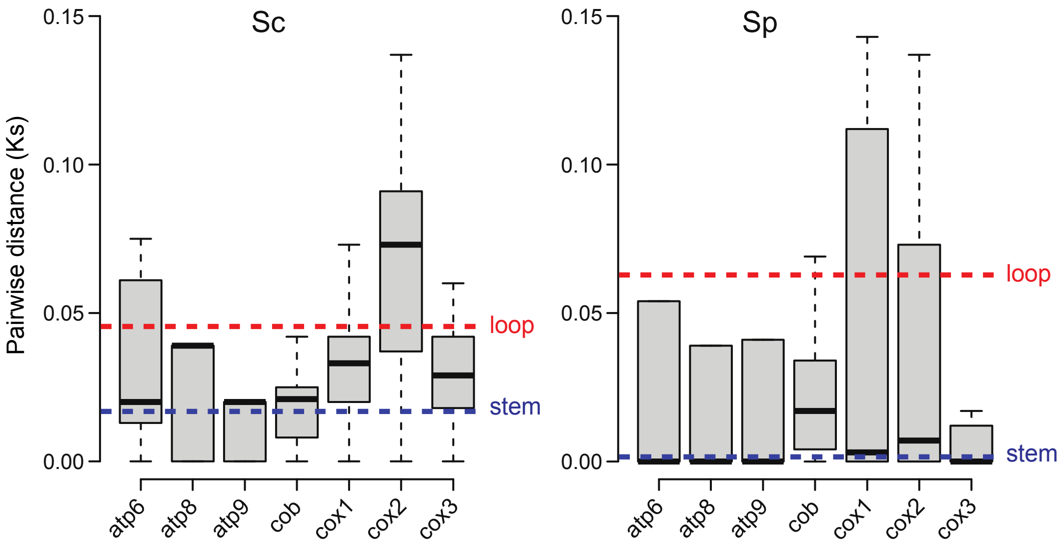

Figure S3 Boxplots of pairwise synonymous nucleotide diversity in seven mitochondrial protein-coding genes, and the average pairwise nucleotide diversity in the GC42 loop region (shown as red lines) as well as the GC42 stem region (shown as blue lines) from *S. cerevisiae* (Sc) and *S. paradoxus* (Sp).

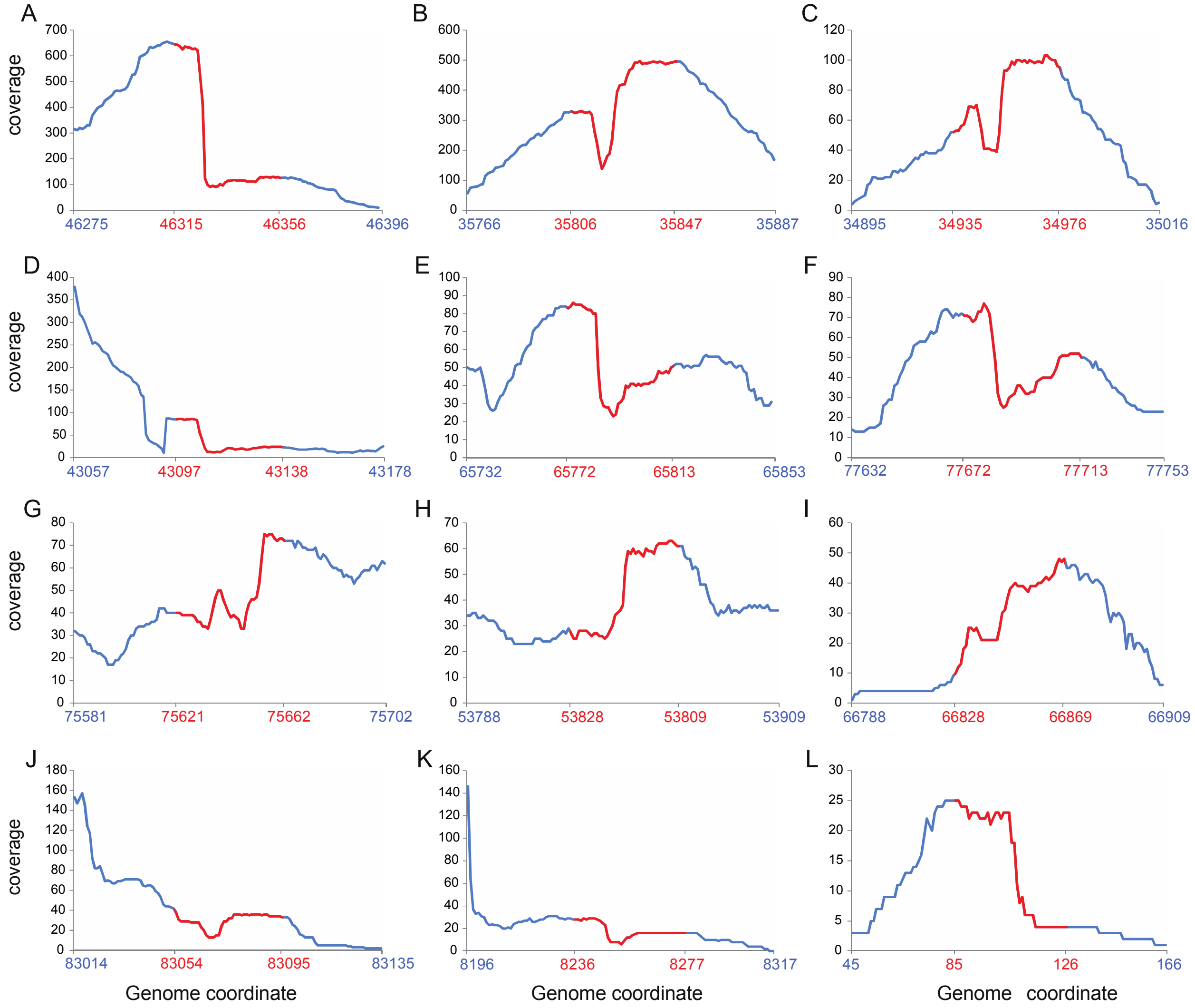

Figure S4 Expression levels of the 12 most highly expressed GC42 sequences and their (40-nucleotide upstream and 40-nucleotide downstream) flanking sequences. The GC42 sequences are ordered by their average expression levels from panels A-L. The GC42 regions are in red, while the flanking regions in blue. Genome coordinates are based on the *S. cerevisiae* S288c reference mitochondrial genome.

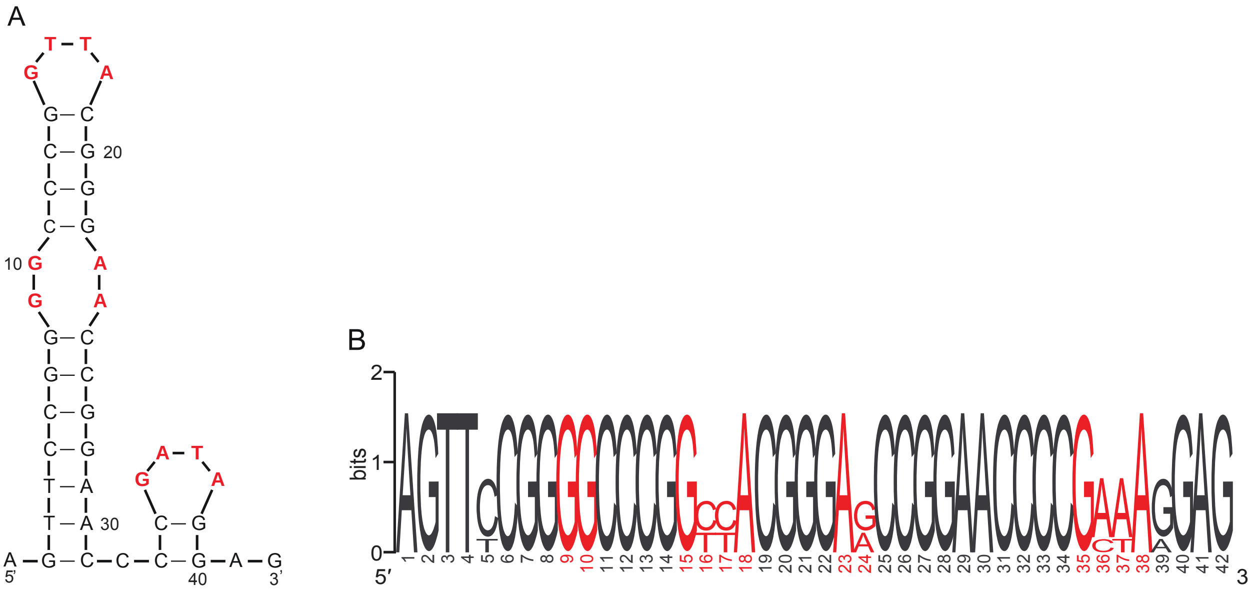

Figure S5 Characteristics of GC42 sequences in *S. paradoxus*. A) Predicted secondary structure of GC42 based on the consensus sequence. The nucleotides in loop regions are in red. B) Sequence logo for all GC42 homologous sequences in *S. paradoxus*. The nucleotides in loop regions are in red.
