# Supplementary material for: A Dynamic Mobile DNA Family in the Yeast Mitochondrial Genome
Source: G3 (Bethesda). 2015 Apr 20;5(6):1273–82. doi: 10.1534/g3.115.017822 (PMC4478555; doi:10.1534/g3.115.017822)
Supplement: Supporting Information [file supp_g3.115.017822_FigureS1.pdf]

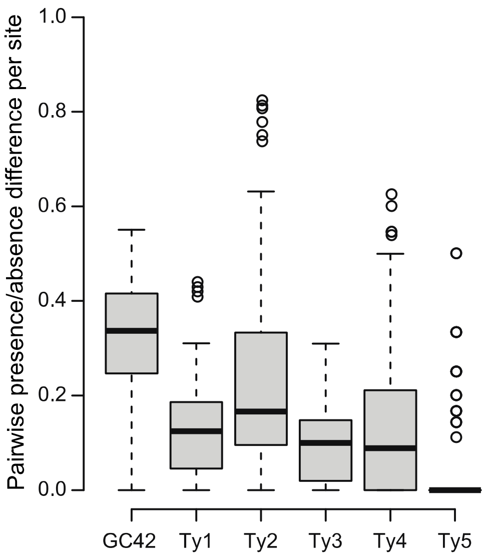

Figure S1 High presence/absence polymorphism in mitochondrial-encoded GC42 in comparison of five nuclear-encoded transposons (Ty1-Ty5) in *S. cerevisiae.* The presence/absence pattern of Ty transposons is obtained from Carr et al. (2012). The pairwise difference is calculated as $\frac{\# different}{\# identical + \# different}$, sites with missing information are excluded from each pairwise comparison.
