# Supplementary material for: A Dynamic Mobile DNA Family in the Yeast Mitochondrial Genome
Source: G3 (Bethesda). 2015 Apr 20;5(6):1273–82. doi: 10.1534/g3.115.017822 (PMC4478555; doi:10.1534/g3.115.017822)
Supplement: Supporting Information [file supp_g3.115.017822_FigureS2.pdf]

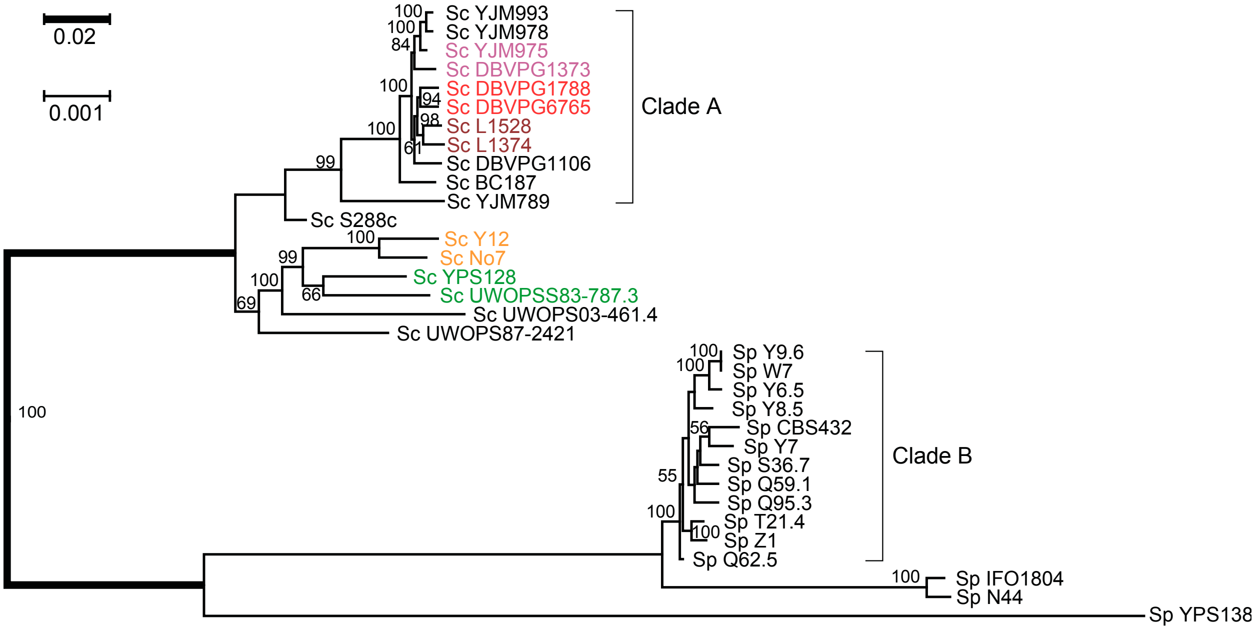

Figure S2 Maximum likelihood phylogeny reconstructed using concatenated sequences of 630 aligned single-copy genes that are universally present in all *S. cerevisiae* and *S. paradoxus* strains. Two closely related clades (clade A and clade B) are subject to further analysis on GC42 turnover (Table 1). Five closely related pairs of genomes were subject to analysis of GC42 density in the indel regions, each pair is shown in a distinct color. YJM975 was chosen to compare against DBPG1373, since no indels >30nt were observed among the mitochondrial genomes of YJM975, YJM993, and YJM978.
