# Supplementary material for: A Dynamic Mobile DNA Family in the Yeast Mitochondrial Genome
Source: G3 (Bethesda). 2015 Apr 20;5(6):1273–82. doi: 10.1534/g3.115.017822 (PMC4478555; doi:10.1534/g3.115.017822)
Supplement: Supporting Information [file supp_g3.115.017822_FigureS3.pdf]

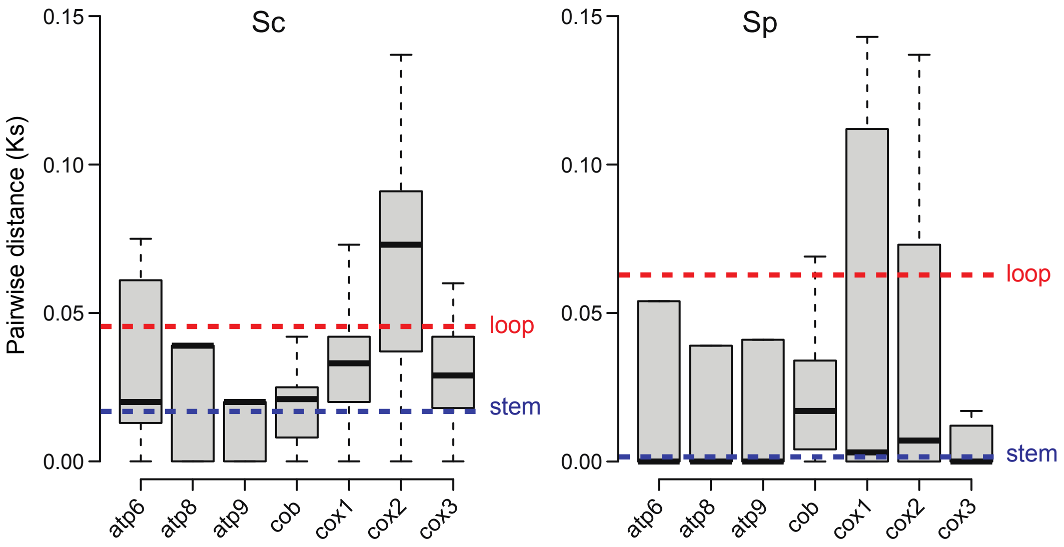

Figure S3 Boxplots of pairwise synonymous nucleotide diversity in seven mitochondrial protein-coding genes, and the average pairwise nucleotide diversity in the GC42 loop region (shown as red lines) as well as the GC42 stem region (shown as blue lines) from *S. cerevisiae* (Sc) and *S. paradoxus* (Sp).
