# Supplementary material for: A Dynamic Mobile DNA Family in the Yeast Mitochondrial Genome
Source: G3 (Bethesda). 2015 Apr 20;5(6):1273–82. doi: 10.1534/g3.115.017822 (PMC4478555; doi:10.1534/g3.115.017822)
Supplement: Supporting Information [file supp_g3.115.017822_FigureS4.pdf]

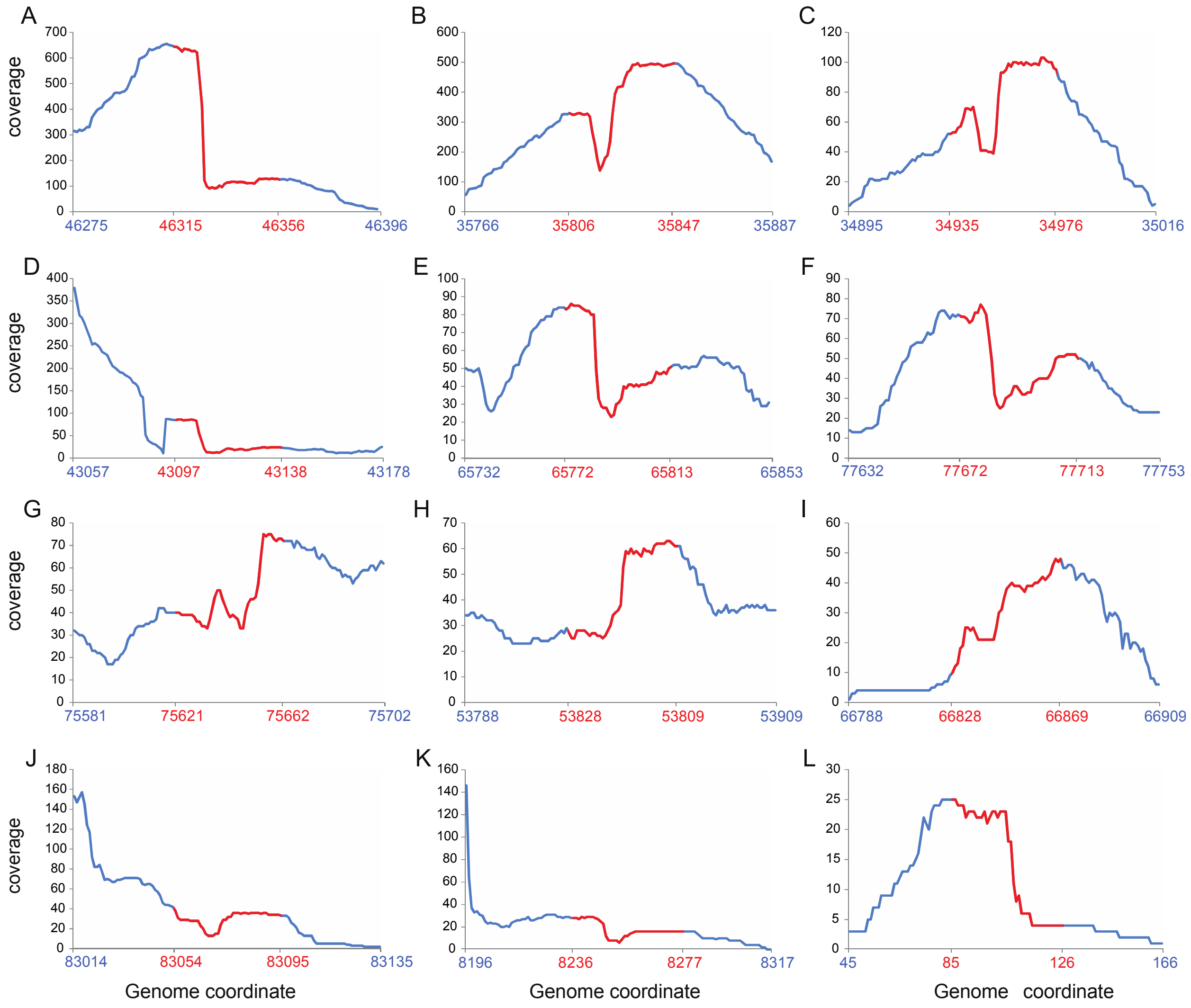

Figure S4 Expression levels of the 12 most highly expressed GC42 sequences and their (40-nucleotide upstream and 40-nucleotide downstream) flanking sequences. The GC42 sequences are ordered by their average expression levels from panels A-L. The GC42 regions are in red, while the flanking regions in blue. Genome coordinates are based on the *S. cerevisiae* S288c reference mitochondrial genome.
