# Supplementary material for: A Dynamic Mobile DNA Family in the Yeast Mitochondrial Genome
Source: G3 (Bethesda). 2015 Apr 20;5(6):1273–82. doi: 10.1534/g3.115.017822 (PMC4478555; doi:10.1534/g3.115.017822)
Supplement: Supporting Information [file supp_g3.115.017822_FigureS5.pdf]

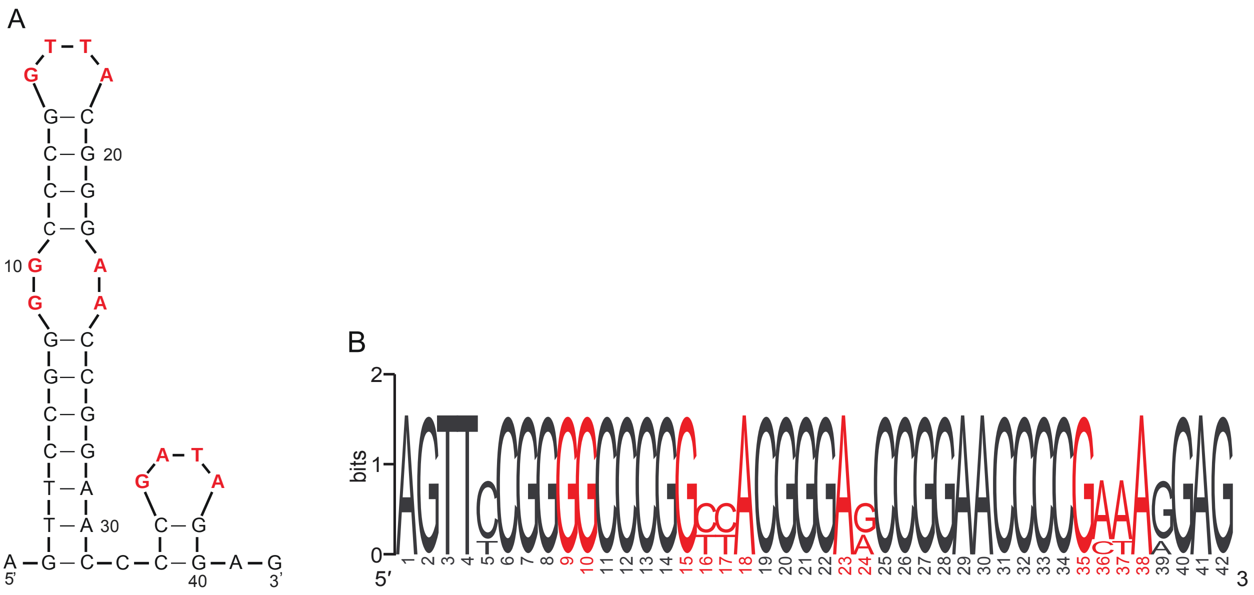

Figure S5 Characteristics of GC42 sequences in *S. paradoxus*. A) Predicted secondary structure of GC42 based on the consensus sequence. The nucleotides in loop regions are in red. B) Sequence logo for all GC42 homologous sequences in *S. paradoxus*. The nucleotides in loop regions are in red.
